# Attitude Moralization Within Polarized Contexts: An Emotional Value-Protective Response to Dyadic Harm Cues

**DOI:** 10.1177/01461672211047375

**Published:** 2021-10-05

**Authors:** Chantal D’Amore, Martijn van Zomeren, Namkje Koudenburg

**Affiliations:** 1 University of Groningen, The Netherlands

**Keywords:** attitude moralization, polarization, dyadic harm, emotions, value protection

## Abstract

Polarization about societal issues involves attitudinal conflict, but we know little about how such conflict transforms into moral conflict. Integrating insights on polarization and psychological value protection, we propose a model that predicts when and how attitude moralization (i.e., when attitudes become grounded in core values) may be triggered and develops within polarized contexts. We tested this model in three experiments (total *N* = 823) in the context of the polarized *Zwarte Piet* (blackface) debate in the Netherlands. Specifically, we tested the hypotheses that (a) situational cues to *dyadic harm* in this context (i.e., an outgroup that is perceived as intentionally inflicting harm onto innocent victims) trigger individuals to moralize their relevant attitude, because of (b) emotional value-protective responses. Findings supported both hypotheses across different regional contexts, suggesting that attitude moralization can emerge within polarized contexts when people are exposed to actions by attitudinal opponents perceived as causing dyadic harm.

Polarization in society implies that different people or groups have conflicting attitudes about political issues. For example, people may disagree about whether abortion should be legalized or penalized, or whether cultural traditions should be changed or protected. Although competition between different ideas in society is important to democratic progress and decision-making (Finkel et al., 2020), attitudinal conflicts in society sometimes intensify and escalate, in particular when they transform into moral conflict (Kovacheff et al., 2018; Mooijman et al., 2018; Skitka & Morgan, 2014; Zaal et al., 2011). Indeed, when both groups in conflict define their position on a political issue in terms of absolute moral “right” versus “wrong” (i.e., as a *moral conviction*), they tend to proactively stand up, or reactively fight, for what (they believe) is of fundamental moral value (Skitka, 2010), with little room for compromises on both sides (e.g., Skitka, 2002; Tetlock et al., 2000; Zaal et al., 2011). This is potentially problematic because it may lead to further dividedness, hostility, and escalation (e.g., Finkel et al., 2020). Therefore, it is important to better understand which ingredients within polarized contexts push attitudinal conflict over the edge, into moral conflict.

Yet, we know little about when and how individuals’ attitudes are pushed into the moral domain—and even less about when and how this occurs within polarized contexts, such as the context of polarized societal debates. Hence, we believe it is essential to better understand when and how *attitude moralization*—defined as the process through which an attitude becomes grounded in one’s core values (Rozin, 1999; Skitka, 2010)—emerges within polarized contexts. Although many have suggested that attitude moralization does not emerge in a social vacuum (Brady et al., 2017; Ellemers et al., 2019; Mooijman et al., 2018; Schein, 2020; van Zomeren, 2016), relatively little is known about the *situational cues* that may trigger it within polarized contexts, and the psychological *process* that may explain its emergence (e.g., Skitka et al., 2018).

In this article, we propose a theoretical model that predicts that individual-level moralization may be triggered within polarized contexts when *situational cues* from a concrete outgroup signal their intention to inflict harm on relevant others (i.e., *dyadic harm*), thereby evoking value-protective *emotions* (Tetlock et al., 2000) that push relevant attitudes into the moral domain (i.e., *attitude moralization*). We test this model in three experiments, in which we manipulate situational cues to dyadic harm by using societal events that could naturally emerge within the context of a specific polarized debate with concretely defined groups: The heated debate in the Netherlands about the cultural figure *Zwarte Piet* (*Black Pete*), which is akin to blackfacing debates in other countries. Specifically, our experimental research was designed to test whether situational cues to dyadic harm serve as potential ingredients within polarized contexts that can trigger psychological moralization via negative moral emotions.

## An Integrative Model of Attitude Moralization in Polarized Contexts

We assume that polarization at the societal level can set the stage for either constructive change or moral conflict to emerge. We define polarization as the existence of conflicting attitudes in society and the structural formation of groups around these attitudes in conflict (Koudenburg & Kashima, 2021; McCoy et al., 2018). Given that attitudinal conflict is not yet of a moral nature, it is possible to resolve and can encourage constructive change through societal discussion and negotiation (e.g., (Finkel et al., 2020; Skitka et al., 2021). However, the more attitudes within both groups get infused with moral meaning, the more the conflict tends to become a self-perpetuating cycle of escalation (Kovacheff et al., 2018; Mooijman et al., 2018; Zaal et al., 2011). For instance, increasing moralization is associated with increasingly negative biases and intolerance toward attitudinal opponents at a psychological level (Finkel et al., 2020; Skitka & Morgan, 2014; Wright et al., 2008) and encourages more extreme and even violent action approaches at a collective level (e.g., Mooijman et al., 2018). Against this backdrop, we assume that the psychological process of attitude moralization on both sides of the debate reflects such a shift from attitudinal to moral conflict.

**Figure 1.**
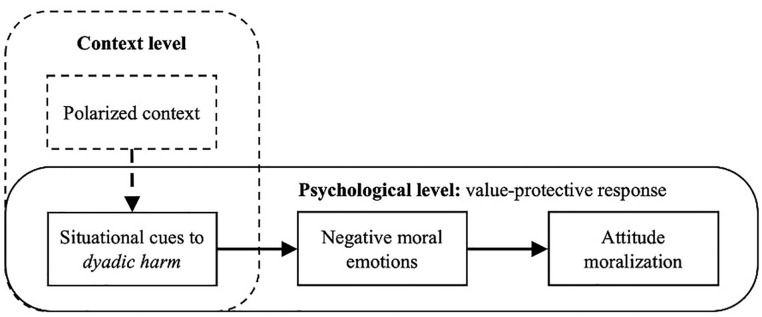
Conceptual model: Dyadic harm as a bridge between the contextual level of polarization and psychological level of attitude moralization. *Note.* The solid boxes and arrows (lower) represent the core variables and relationships of the model that are tested in this research. The dashed boxes and arrow (upper) represent assumptions about the broader background of this research and the relationship that is explored in this research.

We aim to identify this psychological process by integrating *situational triggers* and *psychological mechanisms* for attitude moralization within polarized contexts. Specifically, we propose that the notion of *dyadic harm*, defined as the perceived intentional harm caused to perceived victims (Schein & Gray, 2018), helps to understand *when* moralization is likely to be triggered in these contexts. As for the *how* question, we conceptualize attitude moralization as part of an emotional value-protective response (e.g., Tetlock et al., 2000) to perceived cues to dyadic harm (Schein & Gray, 2018). Figure 1 visualizes our integrative line of thought, illustrating that we use *cues to dyadic harm* as a situational bridge to connect different literatures on polarization, value protection, and attitude moralization.

The model proposes that polarized contexts at the societal level offers situational triggers for psychological moralization because it provides groups in attitudinal conflict, within which group-based behavior emerges that could involve (stronger or weaker) cues to dyadic harm. Indeed, perceiving strong dyadic harm means that the outgroup is intentionally inflicting harm onto innocent victims and is transforming into a dangerous enemy that willfully threatens core values (e.g., equality/tradition). The model therefore proposes that dyadic harm, when perceived, triggers an emotional value-protective response that moralizes individuals’ attitude. As such, attitude moralization is likely to emerge within polarized contexts when situational cues to dyadic harm emerge that trigger negative moral emotions. Below we discuss in more detail how this model integrates insights from different literatures.

### Attitude Moralization as an Emotional Value-Protective Response to Dyadic Harm Cues

Conceptually, our model builds upon the idea of psychological value protection (e.g., Tetlock et al., 2000; see also Fiske & Tetlock, 1997) and combines it with the notion of dyadic harm to better understand how moralization emerges within polarized contexts (Schein & Gray, 2018). Value-protective responses are rooted in a perceived threat to community values, triggering intense emotions like anger and a strong desire to punish the perpetrator as a means to protect these values (McGraw & Tetlock, 2005; for a review, see Kovacheff et al., 2018). Interestingly, although research has suggested that both threatened values and emotional responses may be predictors of attitude moralization, it has not connected these insights with the notion of dyadic harm and against the backdrop of polarized contexts.

Overall, the experience of strong *emotions* like anger or outrage in response to a trigger event seems key to psychological attitude moralization. Specifically, the experience of strong moral emotions toward new information relevant to one’s core values (e.g., Haidt, 2001; Rozin, 1999; Rozin et al., 1999) can push attitudes into the moral domain^1^ (Brandt et al., 2015; Feinberg et al., 2019; see also Kovacheff et al., 2018; Tetlock, 2003). For example, in two different experimental studies, individuals moralized their attitude on a specific issue (i.e., meat consumption, Feinberg et al., 2019; abortion, Wisneski & Skitka, 2017) when they responded with strong moral emotions (i.e., disgust) to morally disruptive videos or images about the relevant issue. Consistent with this, a longitudinal study demonstrated that feelings of anger about the perceived injustices perpetrated by one’s government uniquely and consistently predicted attitude moralization over time (Leal, van Zomeren, Gonzalez, et al., 2020).

In line with the documented importance of moral emotions, attitude moralization is likely tied to the motivation to protect the integrity of one’s core moral values against perceived moral transgressions by others (i.e., *psychological value protection*; for example, Tetlock et al., 2000). For example, recent experimental findings showed that perceived value violations (e.g., blatant discrimination of Muslims or women by the U.S. president) predicted increased attitude moralization about a specific government policy (e.g., the U.S. travel ban; Pauls et al., in press). Comparably, other research found that perceived value violations in the context of gender equality increased attitude moralization specifically when the perpetrating outgroup was perceived as immoral (rather than neutral or moral; Leal, van Zomeren, Gordijn, et al., 2020). These findings fit with the idea that perceived value violations are especially likely to serve as a moralization trigger when these violations are perceived to be *intentional* (as is the case with *dyadic harm*; Schein & Gray, 2018), triggering a stronger psychological need to protect these values (Tetlock, 2003; Tetlock et al., 2000; see also van Zomeren et al., 2018).

Indeed, it is important to differentiate a general notion of harm from *dyadic harm* more specifically, as this latter notion combines subjective perceptions of the *suffering* of victims, the *intentionality* of an actor, and their *dyadic causality* (Schein & Gray, 2018). Within polarized contexts, this subjective element could be of special importance because it implies that moral transgression is difficult to conceptualize in isolation from the social context in which it occurs (e.g., Ellemers & van den Bos, 2012). For instance, recent experimental work (Voelkel & Brandt, 2019) showed that the degree to which a specific action was perceived as involving a *moral* transgression (e.g., violation of the harm/care principle) strengthened when the action involved explicitly specified targets, and in particular, if these targets were relevant to the perceiver (e.g., members of the political party one identifies with; Schein & Gray, 2018). Within polarized contexts, we thus reason that actions taken by an opinion-based *outgroup* can serve as a situational trigger for moralization when this action is perceived as involving strong intentional harm against an opinion-based ingroup.

Correspondingly, it is important to note that we assume that the context of polarization at the societal level features the situational cues that we believe could serve as a trigger for attitude moralization at the individual level. Our model builds on theory and research suggesting that the context of polarization between different groups features ingredients that could trigger moralization, in particular (situations that offer) cues to dyadic ham. This is because the mere existence of two structurally opposing groups in a societal context increases the prevalence of group-based actions aimed at achieving (or preventing) societal change; and in periods of intense polarization (e.g., elections), these actions tend to become increasingly hostile and extreme in nature (e.g., Brady et al., 2020; Iyengar et al., 2012; Levendusky & Malhotra, 2016). This conceptually translates to the idea that these actions are likely to involve strong cues to dyadic harm when polarization in society is salient. For example, when representatives of conflicting groups announce their intention to harm the other group (e.g., the willingness to use violence to prevent societal change)—physically or psychologically—or actually harm members of the other group (e.g., the use of racist slogans). In addition, the likelihood of individuals’ situational exposure to such outgroup actions may be particularly high when actions involve strong cues to dyadic harm. This is because, via social identity processes, strong cues to *us-versus-them* (dyadic) harm are particularly likely to create emotional arousal, capture individuals’ attention, and to motivate individuals to share it with (like-minded) others (Brady et al., 2020). We therefore assume that the context of polarization at the societal level features the situational cues that we believe could serve as a trigger for attitude moralization at the individual level.

## Overview of Hypotheses and Studies

To study the process of moralization *within polarized contexts*, we designed three experiments that test the hypotheses that situational cues to dyadic harm, emerging within polarized contexts, can serve as a trigger for attitude moralization (Hypothesis 1) through the experience of negative moral emotions (Hypothesis 2). We conducted all three experiments within the polarized *Zwarte Piet* context in the Netherlands and used strong (vs. weak) cues to dyadic harm emerging in this debate as a basis for the manipulation. In addition, we measured perceptions of polarization and explored whether variation in polarization may influence this situationally cued moralization process.

To provide some background, the traditional figure *Zwarte Piet* is the black-faced helper of Saint Nicolas (*Sinterklaas*), who are the two main characters in an annual Dutch cultural ritual for children. Although the Dutch have been celebrating *Sinterklaas* for a very long time without any apparent protests or moral condemnation, over the past decade or so, growing numbers of opponents have objected this black-faced characteristic as a form of institutionalized racism and have publicly stood up for seeking social change (e.g., through protesting and seeking to disturb the children’s festivity). In response, *Zwarte Piet* supporters have organized counteractions aimed at protecting their sense of national and cultural identity and tradition (e.g., through blocking highways to stop protesters from reaching their protest site). Given the combined presence of polarized debate and situational cues to intentional harm by these groups in this societal context, ranging from extremely conflict-prone (e.g., use of violence to repress protests) to harmonious approaches (e.g., raising awareness through peaceful demonstrations) that represent stronger and weaker cues to dyadic harm, respectively, we considered this context an excellent opportunity to test our hypotheses.

To do so, we selected a relevant outgroup within this context to be represented in the manipulation, on one hand, and the corresponding participant population, on the other hand. A liberal *Zwarte Piet* activist group (i.e., aimed at instigating change) is well suited as the acting outgroup in the present investigation because this liberal stance was assumed to contrast with the majority opinion in the Netherlands—which tended to be more conservative (as informed by EenVandaag Opiniepanel, 2017^2^). Correspondingly, we recruited participants who have a conservative attitude on the *Zwarte Piet* issue (i.e., opposing change).

Although we focus on the moralization of polarized issues and do not consider non-polarized issues, even within polarized contexts, there could still be a reasonable amount of naturally occurring variance in polarization levels, both at the regional level and at the psychological level. We made use of this variance to explore whether stronger polarization may potentially also have the power to *amplify* individuals’ psychological responses to cues to dyadic harm. Specifically, we aimed to sample from two regions that were assumed to feature moderate and strong levels of attitude polarization (Experiments 1 and 2; June 2019; as informed by public opinion data from EenVandaag Opiniepanel, 2017)^3^ and then sampled broadly across all Dutch regions (Experiment 3; November 2019), to explore whether stronger perceived polarization amplifies value-protective responses to dyadic harm, increasing attitude moralization.

Prior to Experiments 1 and 2, we conducted a pilot study (*N* = 66; February 2019) that showed that our manipulation of dyadic harm in the context of *Zwarte Piet* successfully increased perceptions of dyadic harm and it suggested good construct validity (see Supplementary Materials, pp. 4–6).

## Experiment 1

### Methods

Anonymized data, R-scripts, and materials have been made publicly available online (OSF) at https://osf.io/32fjm/?view_only=ef40ede02acf426fa23187c59753f837.^4^

#### Participants

A representative^5^ sample was recruited through an online provider (*PanelInzicht*). We preselected participants who indicated having a conservative stance on the issue of *Zwarte Piet*, that is, who indicated that they agreed or strongly agreed with the statement “I oppose change in the traditional Zwarte Piet in the Netherlands” (i.e., scores 4–5 on a 5-point scale, ranging from 1 = *strongly disagree* to 5 = *strongly agree*).

Participants were 173 Dutch adults^6^ (58% female; *M*_age_ = 52.5, *SD* = 15.6, range = 18–79; 18% lower educated, 54% middle educated, 27% higher educated), preselected such that they were living in (a) smaller areas within *Friesland* (i.e., the moderately polarized context), and (b) on the countryside (*n* = 70), or in a small city (<100,000 residents; *n* = 103). Participants received credits on their personal account (approximate value of €1.90) for their participation.

#### Procedure

Participants completed an online survey (±15 min), which was available to them for 11 days (June 11–22, 2019). After they read a brief introductory text about the experiment (named “News Perception”), participants reported demographic information and indicated their stance on the *Zwarte Piet* issue, which allowed us to select the appropriate participants (i.e., based on their conservative stance) and to arrive at a representative sample. Following this, they reported on their moral convictions regarding the *Zwarte Piet* issue (pre-measure) and their perceptions of polarization among others around them and in society.

Participants were then randomly allocated to one of the two experimental conditions that were intended to reflect stronger versus weaker cues to dyadic harm—the conflict-prone (*n* = 86) versus harmonious (*n* = 87) action approach, respectively. Subsequently, they reported on two attention checks, their emotional responses, perceived dyadic harm, and perceived immorality regarding the manipulated action approach. Participants then reported on moral conviction again (post-measure), followed by their need to punish this outgroup and their desire for social distance. Finally, they were fully debriefed.

#### Experimental manipulation

The materials used for the manipulation of strong versus weak cues to dyadic harm take the form of news messages that were fabricated by the researchers based on real-life materials from the media. It described a (fictitious) action strategy planned by anti-*Zwarte Piet* activists (i.e., an opinion-based outgroup; McGarty et al., 2009): they strive to implement a carefully designed approach to their activism with the aim of adopting societal alternatives to the traditional *Zwarte Piet* as of the year 2019. We held this aim constant between the two conditions, including the core reason for their activism (i.e., “They insist on changing Zwarte Piet because the racism and discrimination, resulting from the colonial past of the Netherlands, will remain if the black face of Zwarte Piet remains”).

For the manipulation of *cues to dyadic harm* in the conflict-prone (vs. harmonious) condition, we developed two versions of this news message that differ two action approaches. The *harmonious* approach was aimed at initiating harmonious public debate, based on “understanding, tolerance, and solidarity.” In contrast, the *conflict-prone* group was aimed at seeking serious public conflict based on “intimidation, provocation, and, if necessary, violence,” in particular against non-conformers (i.e., *Zwarte Piet* supporters).

#### Measurements

Participants indicated demographic information about their gender, age, and political orientation (10-point scale from 1 = *extremely left-wing oriented* to 10 = *extremely right-wing oriented*). For most measurements reported below, if not stated differently, participants responded on a 5-point scale (from 1 = *not at all* to 5 = *very much*).

##### Dyadic harm

Participants indicated their perceptions of *dyadic harm* by indicating agreement on a three-item measure that taps into the three core elements of dyadic harm (*a* = .87; Schein & Gray, 2018): Namely, the anticipated suffering of victims (“Do you think that this group’s planned actions will leave other people harmed or hurt?”), perceived intention to harm (“Do you think that this group is intending to harm or hurt others with their planned actions?”), and anticipated dyadic causality (“Do you think that this group is responsible, as perpetrator, when other people would feel hurt or harmed by their planned actions?”).

##### Negative moral emotion

Participants reported their emotional responses (i.e., anger, contempt, disgust; Rozin, 1999) toward the conflict-prone [harmonious] group and their statements^7^ (i.e., “To what extent did you feel [emotion] toward the group and their statements?”; *a* = .81).

##### Attitude moralization

Participants indicated, on a validated four-item measure (Skitka & Morgan, 2014; translated to Dutch) the extent to which they held their attitude about the *Zwarte Piet* issue as a moral conviction (e.g., “To what extent is your position on the Zwarte Piet issue a reflection of your core moral beliefs and convictions”; *a_(pre-score)_*= .85; *a_(post-score)_*= .91). Moralization was operationalized as the within-subject increase in moral conviction.

##### Perceived polarization

Participants reported on their perceptions of polarization about the *Zwarte Piet* issue among people in their direct social environment^8^ (i.e., their friends, colleagues, and family). The perceived structural polarization scale (Koudenburg & Kashima, 2021) specifically assesses the perception that groups of people are in direct opposition of one another, and has two items: “In my direct social environment, people stand in direct opposition to one another in how they think about this issue” and “In my direct social environment, people’s viewpoints on the issue are not only divided but also deep-rooted” (*r* = .43).

#### Statistical analysis plan and required sample size

Hypothesis 1 will be tested using an analysis of covariance (ANCOVA) with moral conviction prescores as a covariate and the postscores as the outcome. The ANCOVA approach to the analysis of pretest–posttest designs is statistically preferred over the change-score approach because it enhances statistical power, given that the corresponding assumptions are met (e.g., Oakes & Feldman, 2001; Zientek et al., 2016).^9^ Hypothesis 2 will be tested in two steps, first using the described ANCOVA approach for testing the second direct effect (*b-path*) and then formally testing the indirect effect using Model 4 of the PROCESS macro for SPSS (Hayes, 2012).

As for power analysis, we computed a priori the required sample size for detecting the effect of the conflict-prone (vs. harmonious) manipulation on moralization in our planned experiment, based on the effect size for attitude moralization from a recent longitudinal examination (Feinberg et al., 2019). Specifically, as a liberal indication of the expected effect sizes for moralization, we used the effect size found for the group of participants who showed increased moralization over time (i.e., the “*moralizers*”; small-to-medium effect size, within-subject increase, *f* = 0.188; Cohen, 1992). Using G*Power (version 3.1.9.3), an a priori power analysis for repeated-measures between-factors analysis of variance (ANOVA), with the correlation between repeated measures of *r* = .50 (the default), *Type 1* error probability of *a* = .05, and power of .80, indicated a required sample size of 170 participants. This suggests that our sample size (*N* = 173) is sufficient.

### Results

#### Manipulation check

Replicating the Pilot findings, participants in the conflict-prone (vs. harmonious) condition reported significantly stronger perceptions of dyadic harm, *M_difference_* = 0.99, 95% confidence interval (CI) = [0.67, 1.31], *t*(171) = 6.19, *p* < .001, *d* = 0.94, and immorality, *M_difference_* = 0.95, 95% CI = [0.63, 1.27], *t*(171) = 5.93, *p* < .001, *d* = 0.90; see Table 1 and Table S2 in the Supplementary Materials for an overview of the main variables across all experiments.

**Table 1.** Means (*SD*) by Condition for the Main Variables Across Experiments.

Exp.	Perceived dyadic harm		Negative moral emotion		Moral conviction (pre)		Moral conviction (post)	
	Conflict	Harmonious	Conflict	Harmonious	Conflict	Harmonious	Conflict	Harmonious
Pilot	2.86 (0.73)	1.94 (0.93)	2.34 (1.10)	1.72 (0.89)	—	—	—	—
1	4.12 (0.82)	3.13 (1.24)	3.93 (0.89)	2.78 (1.21)	3.08 (1.11)	3.24 (0.84)	3.21 (0.99)	3.04 (1.18)
2	3.97 (0.97)	2.84 (1.15)	3.75 (1.04)	2.53 (1.28)	3.36 (0.98)	3.05 (1.04)	3.50 (1.01)	2.94 (1.06)
3	3.94 (0.94)	2.93 (1.14)	3.92 (1.02)	2.94 (1.27)	2.84 (1.21)	2.86 (1.25)	3.00 (1.20)	2.84 (1.19)

*Note.* All variables were measured on a 5-point scale (1 = *not at all*, 5 = *completely*).

#### Hypothesis 1: Moralization through a conflict-prone (vs. harmonious) action approach

Analysis of covariance (ANCOVA) with pre-manipulation moral conviction as a covariate indicated a significant between-condition difference on post-manipulation moral conviction, *F*(1,170) = 4.688, *p* = .032; $R_{adjusted}^{2}=.44$ . Regression analysis revealed that, supporting Hypothesis 1, participants who were exposed to the conflict-prone (vs. harmonious) condition showed significantly stronger moralization, *b* = 0.29, 95% CI = [0.03, 0.55]; small-sized effect, $\eta_{\text{p}}^{\text{2}}=.027$ .

#### Hypothesis 2: The mediating role of negative moral emotion

**Figure 2.**
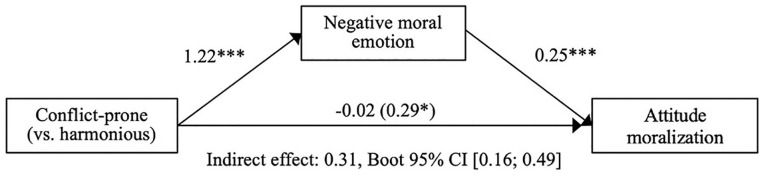
Full mediation model Experiment 1. *Note.* Full mediation model for effects of the conflict-prone (vs. harmonious) group condition on within-subject attitude moralization via negative moral emotion (Experiment 1). Displayed values are unstandardized regression coefficients (upper) and indirect effect corresponding to the bootstrap test of mediation (lower). CI = confidence interval. *p < .05. ***p < .001.

Results supported the hypothesized mediation pattern: first, participants in the conflict-prone (vs. harmonious) condition reported increased negative moral emotion, *b* = 1.22, 95% CI = [0.92, 1.51], *t*(170) = 8.176, *p* < .001; large-sized effect, *$\eta_{\text{p}}^{\text{2}}=.282$ *. Second, negative moral emotion was a significant and positive predictor for moralization when it was added to the model with the condition, *b* = 0.25, 95% CI = [0.12, 0.38], *t*(169) = 3.846, *p* < .001; medium-sized effect, $\eta_{\text{p}}^{\text{2}}=.080$ , and the direct effect of the conflict-prone (vs. harmonious) condition reduced to non-significance, *b* = −0.02, 95% CI = [−0.32, 0.28], *t*(169) = −0.136, *p* = .892. Finally, bootstrapped mediation analysis test showed that the indirect effect of the condition on moralization via negative moral emotion was positive and significant, *indirect effect* = 0.31, Bootstrapped 95% CI = [0.16, 0.49]. This supports our hypothesis that conflict-prone (vs. harmonious) actions by an opposing action group increased participants’ attitude moralization about the *Zwarte Piet* issue via the experience of negative moral emotions (*robust $R_{adjusted}^{2}=.48$ *; see Figure 2 for a visualization).^10^

### Discussion Experiment 1

Experiment 1 found first experimental support for our hypotheses among a sample of participants from a region in the Netherlands that was assumed to feature a moderately polarized regional context. The findings showed that our manipulation of strong dyadic harm triggered a process of attitude moralization via the experience of negative moral emotions. The aim of Experiment 2 was to directly replicate Experiment 1 with participants from a region in the Netherlands that was assumed to feature a more strongly polarized regional context regarding the *Zwarte Piet* issue.

## Experiment 2

### Methods

#### Participants

The sample included 155 Dutch adults (48% female; *M*_age_= 52.94, *SD* = 16.6, range = 18–85; 23% lower educated, 47% middle educated, 30% higher educated), preselected such that they were living in (a) large cities (>100,000 residents) and (b) in the Dutch provinces *Noord-Holland* (*n* = 44) or *Zuid-Holland* (*n* = 111; that is, the relatively polarized regional context).

#### Measures and procedure

The same measurements as in Experiment 1 were used: moral conviction was measured both prior to (*a* = .84) and after the manipulation (*a* = .87). Furthermore, participants completed the same measures of negative moral emotion (*a* = .85), perceived dyadic harm (*a* = .88), immorality (*a* = .91), and perceived polarization (*r* = .48) Participants were randomly allocated to either the conflict-prone (*n* = 82) or harmonious condition (*n* = 73).

### Results

#### Manipulation check

The manipulation was successful: as in Experiment 1, participants in the conflict-prone (vs. harmonious) condition reported significantly stronger perceptions of dyadic harm, *M_difference_* = 1.13, 95% CI = [0.79, 1.47]; *t*(153) = 6.64, *p* < .001, *d* = 1.07, and immorality, *M_difference_* = 1.33, 95% CI = [1.00, 1.66]; *t*(153) = 7.84, *p* < .001, *d* = 1.29.

#### Explorative comparison of local polarization levels between Experiments 1 and 2

We explored whether average perceived polarization in individuals’ local environment differed between Experiments 1 and 2. However, we found no clear support for the proposed differences. Specifically, we found no differences between participants’ perceived *structural polarization* in their direct social environment, Exp 1: *M* = 2.05, *SD* = 1.02; Exp 2: *M* = 2.17, *SD* = 1.07; *t*(326) = 1.08, *p =* .281, which was our core measure for perceived polarization in participants’ regional contexts. Thus, there is no conclusive evidence supporting the assumption of stronger levels of regional polarization in the sample of Experiment 2 compared with Experiment 1. For this reason, we could not use a regional comparison between the two experiments as a means to explore the potential role of variance in polarization levels in a meaningful way. Instead, in Experiment 3, we conduct the same study in a broad national sample with sufficient power to explore a potential amplification effect of naturally occurring variance in *individual perceptions* of polarization.

#### Hypothesis 1: Moralization through a conflict-prone (vs. harmonious) action approach

Replicating Experiment 1, we found a significant between-condition difference on moralization, *F*(1,152) = 7.991, *p* = .005; $R_{adjusted}^{2}=.49$ . In line with Hypothesis 1, participants in the conflict-prone (vs. harmonious) condition reported increased moralization (*b* = 0.35, 95% CI = [0.11, 0.60]; small-sized effect, $\eta_{\text{p}}^{\text{2}}=.050$ ).

#### Hypothesis 2: The mediating role of negative moral emotion

**Figure 3.**
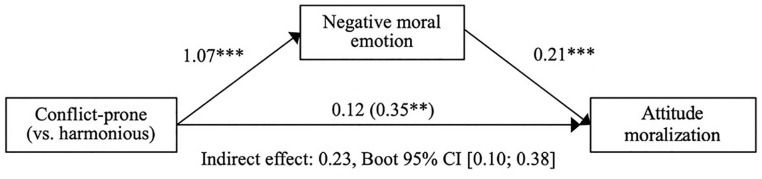
Full mediation model Experiment 2. *Note.* Full mediation model for effects of the conflict-prone (vs. harmonious) group condition on within-subject attitude moralization via negative moral emotion (Experiment 2). Displayed values are unstandardized regression coefficients (upper) and indirect effect corresponding to the bootstrap test of mediation (lower). CI = confidence interval. **p < .01. ***p < .001.

Replicating Experiment 1, participants in the conflict-prone (vs. harmonious) group condition reported increased negative moral emotion, *b* = 1.07, 95% CI - [0.73, 1.40], *t*(152) = 6.327, *p* < .001; large-sized effect, $\eta_{\text{p}}^{2}=.208$ . Moreover, negative moral emotion had a significant and positive effect on moralization when it was added to the model, *b* = 0.21, 95% CI = [0.10, 0.33], *t*(151) = 3.723, *p* < .001; medium-sized effect, $\eta_{\text{p}}^{2}=.084$ , and the direct effect of the conflict-prone (vs. harmonious) condition reduced and became non-significat, *b* = 0.12, 95% CI = [−0.14, 0.39], *t*(151) = 0.919, *p* = .359. Again as expected, the indirect effect via negative moral emotion on moralization was positive and significant, *indirect effect* = 0.23, Bootstrapped 95% CI = [0.10, 0.38]. This suggests that when the group was conflict-prone (vs. harmonious), participants increased in their moral conviction via the experience of negative moral emotions ( $R_{adjusted}^{2}=.53$ ; see Figure 3 for a visualization).^11^

### Discussion Experiment 2

Experiment 2 replicated experimental support for both hypotheses; this time among participants from a region in the Netherlands that was expected (but not found) to feature a more strongly polarized regional context. In this context, the manipulation of dyadic harm also led to increased attitude moralization via the experience of negative moral emotions. This suggests that perceiving dyadic harm can be a crucial trigger for attitude moralization, that strong emotions help push attitudes into the moral domain, and that a background of polarized debate on the issue offer situational triggers for attitude moralization to emerge.

Experiments 1 and 2 show that the proposed attitude moralization process occured across different regional contexts. However, because our study revealed no reliable differences between the regions’ polarization levels, we could not explore a potential *amplification by polarization effect.* To address this issue and further replicate our core findings across the Netherlands, Experiment 3 includes a broad sample of participant from across all different regions (provinces) in the Netherlands that allows us to capture *individual* variation in perceived polarization levels in society.

## Experiment 3

### Methods

#### Participants

Participants were recruited via an online provider (*PanelInzicht*) and received monetary compensation for their participation. As in Experiments 1 and 2, we preselected participants who indicated that they opposed change in the traditional *Zwarte Piet*, but we slightly adapted this measure for two reasons. First, we made the statement neutral as to make it more intuitive to answer (i.e., “How do you think about changing the traditional Zwarte Piet in the Netherlands?”). Second, we broadened the answer scale to a 7-point scale (1 = *very strongly support*, to 4 = *neither support nor oppose*, to 7 = *very strongly oppose*) to allow for more variation on this measure within our sample. Correspondingly, we preselected participants who selected one of the response options from *oppose* to *very strongly oppose* (i.e., scores 5–7).

Participants are 495 Dutch adults^12^ (55% females; *M*_age_ = 48.36, *SD =* 15.6, range = 18–84; 14% lower educated, 52% middle educated, 34% higher educated). This sample is approximately representative in terms of residence in urban/suburban/rural areas and across the 12 Dutch provinces (see Supplementary Materials for a full sample description).

#### Procedure

Participants were again randomly assigned to either the conflict-prone (*n* = 248) or the harmonious (*n* = 247) condition. The procedure of Experiment 3 was similar to Experiments 1 and 2, with the exception of some minor cosmetic changes in the phrasing of the manipulation, and the inclusion of larger scales for perceptions of polarization (see below) to increase the reliability of these measures.

#### Measurements

The measurements assessed in Experiment 3 closely resembled those used in Experiments 1 and 2. Participants completed the four-item measure of moral conviction, prior to (*a* = .89) and after the manipulation (*a* = .90). Furthermore, they completed the same measures of negative moral emotions (*a* = .85), dyadic harm (*a* = .85), and immorality (*a* = .88).

##### Perceived polarization

Perceived polarization was assessed with a five-item scale for perceived structural polarization (Koudenburg & Kashima, 2021), tailored to assess perceptions about both the Dutch population and participants’ direct (local) social environment, *a_(NL)_*= .75, *a_(local)_* = .83.

#### Power analysis

We followed the same analysis plan as in Experiments 1 and 2. In addition, we explore the potential amplification effect of polarization by means of testing interactions between individual perceptions of polarization and moralization and the conflict-prone (vs. harmonious) condition.

For the a priori power analyses, we computed the required sample size to detect medium-sized attenuated^13^ interaction effects (i.e., *f* = 0.125; Blake & Gangestad, 2020). Using G*Power (version 3.1.9.3), a priori power analysis for an ANCOVA, numerator *df* = 1, two groups, *Type 1* error probability of *a* = .05, and power of .80, indicated a required sample size of 505 participants. We aimed at collecting a conservative total of 550 participants to allow for the exclusion of participants who failed the attention check.

### Results

#### Manipulation checks

Replicating Experiments 1 and 2, independent sample *t*-tests showed that participants in the conflict-prone (vs. harmonious) condition perceived stronger dyadic harm, *M_difference_* = 1.01, 95% CI = [0.83, 1.20], *t*(493) = 10.63, *p* < .001, *d* = 0.96, and immorality, *M_difference_* = 1.04, 95% CI = [0.85, 1.23], *t*(493) = 10.66, *p* < .001, *d* = 0.96.

#### Hypothesis 1: Moralization through a conflict-prone (vs. harmonious) group action

An ANCOVA with pre-moral conviction scores as a covariate indicated a significant between-condition difference on post-moral conviction scores, *F*(1,492) = 5.087, *p* = .025; $R_{adjusted}^{2}=.51$ . Regression analysis revealed that, supporting Hypothesis 1, participants who were exposed to the conflict-prone (vs. harmonious) manipulation reported significantly stronger moralization, *b* = 0.17, 95% CI = [0.02, 0.32]; small-sized effect, $\eta_{\text{p}}^{\text{2}}=.010$ ).

#### Hypothesis 2: The mediating role of negative moral emotions

**Figure 4.**
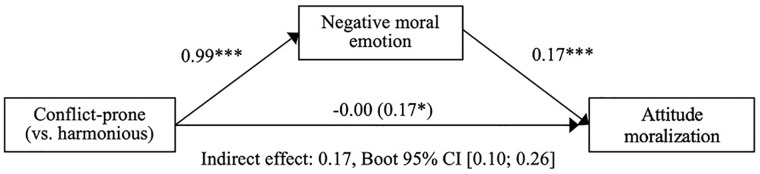
Full mediation model Experiment 3. *Note.* Full mediation model for effects of the conflict-prone (vs. harmonious) group condition on within-subject attitude moralization via negative moral emotion (Experiment 3). Displayed values are unstandardized regression coefficients (upper) and indirect effect corresponding to the bootstrap test of mediation (lower). CI = confidence interval. *p < .05. ***p < .001.

As in Experiments 1 and 2, participants in the conflict-prone (vs. harmonious) condition reported increased negative moral emotion, *b* = 0.99, 95% CI = [0.79, 1.18], *t*(492) = 9.940, *p* < .001; small-sized effect, $\eta_{\text{p}}^{\text{2}}=.053$ . Furthermore, negative moral emotion positively predicted moralization when it was added to the model with the condition, *b* = 0.17, 95% CI = [0.11, 0.24], *t*(491) = 5.218, *p* < .001; medium-sized effect, $\eta_{\text{p}}^{\text{2}}=.062$ , whereas the direct effect of the conflict-prone (vs. harmonious) manipulation was not significant anymore, *b* = −0.00, *t*(491) = −0.02, *p* = .983. In addition, robust mediation analyses showed that the indirect effect via negative moral emotion on moralization was positive and significant, *indirect effect* = 0.17, Bootstrapped 95% CI = [0.10, 0.26]. This suggests that when the action was conflict-prone (vs. harmonious), participants increased in their moral conviction against change because they experienced stronger moral emotions ( $R_{adjusted}^{2}=.54$ ; see Figure 4 for a visualization).^14^

#### Exploration of perceived polarization effects

Experiment 3 additionally explored whether individuals’ increased perceptions of polarization may amplify moralization in response to observing situational cues to dyadic harm. To do so, we used a mixed-effects model with perceived polarization at Level 2 (subject level) and time (pre-measure and post-measure moral conviction) at Level 1 to test for potential interaction between perceived polarization in society and the *conflict-prone* (vs. *harmonious*) group condition on within-subject moralization, but no support was found for this interaction, *F*(1,491) = 0.512, *p* = .475. This suggests that the direction and size of the effect of the dyadic harm manipulation on moralization did not differ as a function of perceived polarization.^15^ We interpret this finding further in the “General Discussion” section.

A further exploration of the potential role of perceived polarization nevertheless showed an interesting pattern of *additive effects* (across studies). Specifically, a mixed-effects model with the experimental condition and perceived polarization as a between-subject predictor showed a positive effect of perceived polarization on individuals’ moral conviction, over and beyond the effect of the condition, across all three experiments: Experiment 1, *F*(1,170) = 3.991, *p* = .047; *B* = 0.16, 95% CI = [0.00, 0.32], Experiment 2, *F*(1,152) = 11.692, *p* = .001; *B* = 0.29, 95% CI = [0.13, 0.46], and Experiment 3, *F*(1,492) = 38.223, *p* < .001; *B* = 0.41, 95% CI = [0.28, 0.54]. This suggests that, over and beyond the effect of the experimental condition, there is a positive association between individuals’ perceptions of polarization in society about the issue of *Zwarte Piet* and the general strength of their moral conviction about this specific issue, which we also discuss further below.

## General Discussion

We examined when and how attitude moralization may be triggered and emerge within polarized contexts. As formalized in our integrative model (Figure 1), we expected that situational cues to dyadic harm can trigger attitude moralization (Hypothesis 1), because of the emotional value-protective responses evoked by such situational cues (Hypothesis 2). Across three experiments conducted in different contexts, we found consistent evidence in line with both hypotheses. Thus, our findings demonstrate that attitude moralization can emerge in situations with strong cues to dyadic harm against the backdrop of polarized debate. Moreover, the process of moralization seems, within polarized societal contexts at least, emotive and value-protective. This suggests that attitude moralization in polarized contexts can be driven by the powerful motivation to protect societal values from perceived attacks by dangerous outgroups and thus underscores the potential of situational cues to dyadic harm (Schein & Gray, 2018) for triggering this psychological process within polarized contexts.

### Theoretical and Practical Implications

The findings of this research build on and extend theorizing and research on moral psychology, value protection, and societal polarization in various ways. Below we discuss six specific implications of our findings.

#### Harm as dyadic

First, our findings suggest that a focus on *dyadic* harm may be key to understanding triggers for attitude moralization within polarized contexts. Although most researchers have assigned the general concept of harm a central role in theory on moral judgments (e.g., Kohlberg, 1969; Piaget, 1965; Rozin & Singh, 1999; Turiel, 2006), no previous research on moralization has specifically focused on the dyadic element of harm within polarized contexts. The few empirical studies that examined the role of harm as a general (utilitarian) predictor in the process of attitude moralization about a polarized issue (Brandt et al., 2015; Wisneski & Skitka, 2017) did not find clear support for its predictive power. Interestingly, our consistent finding that strong cues to dyadic harm served as a situational trigger for attitude moralization adds to this literature by suggesting that for understanding moralization triggers within polarized contexts, it is important to understand when people perceive harm as more dyadic (in this case, when a concrete outgroup is perceived as intentionally harming innocent [ingroup] victims). Indeed, we suggest that, in polarized contexts at least, harm could trigger attitude moralization when it is perceived to be dyadic—that is, intentionally harmful. This implies that researchers interested in predicting attitude moralization within polarized contexts should consider conceptualizing and measuring harm as dyadic.

#### Emotional value-protective responses

Second, our approach to moralization supports the idea that the psychological process of attitude moralization is highly *emotional*. Corroborating previous research, we found that the experience of negative moral emotions served as the psychological mechanism driving attitude moralization (Brandt et al., 2015; Feinberg et al., 2019; Leal, van Zomeren, Gonzalez, et al., 2020; Rozin et al., 1999; Wisneski & Skitka, 2017). Moreover, previous research found that emotions lead to attitude moralization only when these emotions arise consciously and are relevant to the topic at hand (i.e., *integral affect*), suggesting that emotional responses alone are not sufficient for moralization to occur (Wisneski & Skitka, 2017). We add to this by suggesting that within polarized contexts, such negative moral emotions should be particularly likely to bring about attitude moralization when they occur as value-protective responses to perceived dyadic harm by a concrete outgroup. Hence, rather than contrasting psychological explanations based on emotions against those based on beliefs about harm and welfare (see McAuliffe, 2019), we suggest these are not mutually exclusive (Schein & Gray, 2018). In fact, we believe that negative moral emotions explained attitude moralization as triggered by perceived dyadic harm in our studies because these responses serve to protect core values from threat or attack.

#### Attitude moralization within polarized contexts

Third, our findings support the assumption that polarized contexts offer features that enable situational triggers for attitude moralization, specifically cues to dyadic harm. This is because the context of societal polarization, in particular the structural formation of groups around conflicting attitudes, tends to feature increasingly hostile and conflict-prone group actions that are likely to involve meaningful cues to dyadic harm and thus potential triggers for moralization (e.g., McCoy et al., 2018). Although the current research cannot determine the extent to which cues to dyadic harm become more pronounced as issues become more polarized, our findings make the first step to demonstrate that *when* situational cues to dyadic harm arise in a polarized context, these can serve as situational triggers for attitude moralization.

Fourth, against this backdrop it is important to note that although we found little evidence for an amplifying effect of polarization on attitude moralization, we believe it is too soon to draw a firm conclusion; particularly because this set of studies suggested a generally positive effect of individuals’ perceptions of polarization on the strength of their moral convictions at the between-subject level. One important limitation, however, was that perceived polarization was only measured once and therefore cannot yet inform us about the potential role that *within-individual increases* in perceptions of polarization (rather than between-subject differences) may play within the psychological process of moralization. Nevertheless, a recent study that methodologically was better suited to test this hypothesis employed a four-wave longitudinal study in the context of the 2020 U.S. presidential election and found that increased perceptions of polarization within an individual amplified their perception of dyadic harm cues over time and in this way predicted their attitude moralization over time (D’Amore et al., 2021). Clearly, more research is needed to better understand the polarization-moralization link in more detail.

Fifth, our model and findings have the potential to further integrate structural, situational, and psychological factors involved in the process of attitude moralization. Indeed, previous work focused on the moralization of either specific actions, or (social) entities, or attitudes—largely independent from each other (Rhee et al., 2019). Yet, our model suggests that these three processes may, under some conditions, be psychologically intertwined—especially within polarized contexts. As illustrated by our findings, the perception that a situational *action* involves dyadic harm—and thus moral wrongness (Schein & Gray, 2018)—can serve as a moralization trigger for individuals’ relevant *attitude*, likely via the moralization of the responsible *outgroup* (Crimston et al., 2018; that is, entity moralization; Rhee et al., 2019) that connects this situational action with the relevant attitude. We explain this by suggesting that in polarized contexts with *structural* conflict between different groups, the situational salience of harmful and immoral intentions by a concrete outgroup against a concrete and relevant ingroup implies a psychological transformation of “them” into a dangerous societal enemy that threatens “our” moral values. This suggests that it is important to contextualize general predictors for moralization (Schein, 2020; van Zomeren, 2016) if we want to understand its potential triggers in natural contexts.

#### Moral polarization

Finally, our research assumes, but was not designed to show, that polarization and attitude moralization can have both positive and negative consequences. Although the moralization of attitudes may help enforce progressive social change (e.g., Feinberg et al., 2019; Skitka & Bauman, 2008), it may also escalate existing disagreements between different groups in society into violence (Mooijman et al., 2018; Rai & Fiske, 2012)—both of which can be perceived as desirable or undesirable. This is precisely, we believe, why it is so important to better understand attitude moralization against the backdrop of polarized contexts. Previous research has documented the potentially detrimental consequences of psychological moralization, including the idea that moral conflicts between groups (cf. *moral polarization*) may be a root cause of pressing societal problems (for a review, see Kovacheff et al., 2018). For instance, people who are perceived to transgress moral boundaries are considered not only wrong but inherently dangerous: those who reject “our” moral worldview are often vilified and even dehumanized (e.g., Brady et al., 2020; Garrett & Bankert, 2018; Skitka & Morgan, 2014). Taken together with the idea that attitudinal polarization in society offers situational ingredients that could trigger attitude moralization, specifically cues to dyadic harm, this points at a mutually reinforcing cycle between societal-level polarization and individual-level moralization; a combination that may be a recipe for social change on one hand but civil war on the other hand (e.g., McCoy et al., 2018). As such, attitude moralization is a potentially explosive tool to yield in polarized contexts and needs to be better understood to be used responsibly.

### Limitations and Future Directions

This set of studies also has at least four limitations. First, all studies used the same context: We chose the *Zwarte Piet* context because it was highly relevant for our Dutch participants and replicated it within this context to assess the robustness (replicability) of the found effects. However, this may have come at the cost of reduced external validity: although we showed that the key findings can be generalized across different regional contexts within society, its generalizability across different societal contexts is clearly a direction for future research.

Second, it is possible that results were influenced by the relatively short period of time (approximately 10 min) between the two repeated measures of moral conviction that together constituted our assessment of attitude moralization. Although we do not have clear reasons to expect that increased moral conviction, if induced, would last only temporarily (Feinberg et al., 2019), this question is worthy of future research.

Third, we believe that the measurement of perceived polarization can improve in future studies. Although we distinguished between individuals’ perceptions of society at large and of their everyday social environment in the measures used for perceived polarization, it remains an open question which (type of) people were mostly taken into account when participants were asked about their direct social environment. Future research can improve the measurement of perceived polarization regarding these different social settings (e.g., discussion networks, close vs. distant others; Ellemers, & van den Bos, 2012; Morey et al., 2012), to better explore its potential amplifying power.

Finally, future experimental research could enhance our understanding of the relationship between perceived polarization and attitude moralization based on a within-subject design that includes a range of different cues to dyadic harm (e.g., by varying the salience of one vs. two groups; varying the ambiguity of harmful intentions), which could help to identify specific conditions under which perceived polarization does or does not strengthen perceptions of dyadic harm and attitude moralization.

### Conclusion

Little research to date has examined when and how attitude moralization may occur within polarized contexts. Our research corroborated the integrative model proposed in this paper by showing that, across three experiments, situational cues to dyadic harm within the context of a polarized debate served as situational triggers for attitude moralization. This effect was explained by an emotional value-protective response to these cues within polarized contexts. These findings thus suggest it is fruitful to move beyond the common “isolated-individual” approach commonly taken in this literature (e.g., Ellemers, 2017; Schein, 2020; van Zomeren, 2016) by means of contextualizing potential psychological triggers for attitude moralization. Moreover, these findings are socially consequential: If outgroup actions that involve strong dyadic harm can serve as a trigger for attitude moralization among their opponents, then the repeated emergence of cues to dyadic harm between groups may serve to moralize public opinion on both sides of the debate over time, with growing moral conflict as a societal consequence. We therefore hope that a more integrative understanding of the psychology of attitude moralization will help individuals and societies to avoid such a consequence.

## Supplemental Material

sj-docx-1-psp-10.1177_01461672211047375 – Supplemental material for Attitude Moralization Within Polarized Contexts: An Emotional Value-Protective Response to Dyadic Harm Cues Click here for additional data file. Supplemental material, sj-docx-1-psp-10.1177_01461672211047375 for Attitude Moralization Within Polarized Contexts: An Emotional Value-Protective Response to Dyadic Harm Cues by Chantal D’Amore, Martijn van Zomeren and Namkje Koudenburg in Personality and Social Psychology Bulletin

sj-docx-2-psp-10.1177_01461672211047375 – Supplemental material for Attitude Moralization Within Polarized Contexts: An Emotional Value-Protective Response to Dyadic Harm Cues Click here for additional data file. Supplemental material, sj-docx-2-psp-10.1177_01461672211047375 for Attitude Moralization Within Polarized Contexts: An Emotional Value-Protective Response to Dyadic Harm Cues by Chantal D’Amore, Martijn van Zomeren and Namkje Koudenburg in Personality and Social Psychology Bulletin This article is distributed under the terms of the Creative Commons Attribution 4.0 License ( http://www.creativecommons.org/licenses/by/4.0/) which permits any use, reproduction and distribution of the work without further permission provided the original work is attributed as specified on the SAGE and Open Access pages ( https://us.sagepub.com/en-us/nam/open-access-at-sage).

sj-r-6-psp-10.1177_01461672211047375 – Supplemental material for Attitude Moralization Within Polarized Contexts: An Emotional Value-Protective Response to Dyadic Harm Cues Click here for additional data file. Supplemental material, sj-r-6-psp-10.1177_01461672211047375 for Attitude Moralization Within Polarized Contexts: An Emotional Value-Protective Response to Dyadic Harm Cues by Chantal D’Amore, Martijn van Zomeren and Namkje Koudenburg in Personality and Social Psychology Bulletin This article is distributed under the terms of the Creative Commons Attribution 4.0 License ( http://www.creativecommons.org/licenses/by/4.0/) which permits any use, reproduction and distribution of the work without further permission provided the original work is attributed as specified on the SAGE and Open Access pages ( https://us.sagepub.com/en-us/nam/open-access-at-sage).

sj-rdata-3-psp-10.1177_01461672211047375 – Supplemental material for Attitude Moralization Within Polarized Contexts: An Emotional Value-Protective Response to Dyadic Harm Cues Click here for additional data file. Supplemental material, sj-rdata-3-psp-10.1177_01461672211047375 for Attitude Moralization Within Polarized Contexts: An Emotional Value-Protective Response to Dyadic Harm Cues by Chantal D’Amore, Martijn van Zomeren and Namkje Koudenburg in Personality and Social Psychology Bulletin This article is distributed under the terms of the Creative Commons Attribution 4.0 License ( http://www.creativecommons.org/licenses/by/4.0/) which permits any use, reproduction and distribution of the work without further permission provided the original work is attributed as specified on the SAGE and Open Access pages ( https://us.sagepub.com/en-us/nam/open-access-at-sage).

sj-rdata-4-psp-10.1177_01461672211047375 – Supplemental material for Attitude Moralization Within Polarized Contexts: An Emotional Value-Protective Response to Dyadic Harm Cues Click here for additional data file. Supplemental material, sj-rdata-4-psp-10.1177_01461672211047375 for Attitude Moralization Within Polarized Contexts: An Emotional Value-Protective Response to Dyadic Harm Cues by Chantal D’Amore, Martijn van Zomeren and Namkje Koudenburg in Personality and Social Psychology Bulletin This article is distributed under the terms of the Creative Commons Attribution 4.0 License ( http://www.creativecommons.org/licenses/by/4.0/) which permits any use, reproduction and distribution of the work without further permission provided the original work is attributed as specified on the SAGE and Open Access pages ( https://us.sagepub.com/en-us/nam/open-access-at-sage).

sj-rdata-5-psp-10.1177_01461672211047375 – Supplemental material for Attitude Moralization Within Polarized Contexts: An Emotional Value-Protective Response to Dyadic Harm Cues Click here for additional data file. Supplemental material, sj-rdata-5-psp-10.1177_01461672211047375 for Attitude Moralization Within Polarized Contexts: An Emotional Value-Protective Response to Dyadic Harm Cues by Chantal D’Amore, Martijn van Zomeren and Namkje Koudenburg in Personality and Social Psychology Bulletin This article is distributed under the terms of the Creative Commons Attribution 4.0 License ( http://www.creativecommons.org/licenses/by/4.0/) which permits any use, reproduction and distribution of the work without further permission provided the original work is attributed as specified on the SAGE and Open Access pages ( https://us.sagepub.com/en-us/nam/open-access-at-sage).

## Notes

1. This is a psychological phenomenon called moral piggybacking (Feinberg et al., 2019; Rozin, 1999).

2. Note that public opinion has gradually been shifting: support for change in Zwarte Piet increased after the first two experiments were conducted in June 2019.

3. Experiment 1 is sampled from a region where the outgroup’s anti-Zwarte Piet stance is generally rejected (i.e., rural areas in Friesland; moderate polarization), and Experiment 2 from a region where opinion distribution revolves around the 50–50 mark (i.e., urban areas in the Randstad; strong polarization).

4. No studies in this manuscript were preregistered.

5. The sample was representative for the Dutch adult population in terms of gender, age, and educational level, in line with the most recent CBS data (Central Bureau for Statistics, 2019).

6. Participants who did not correctly select the manipulated action approach (i.e., attention check) were excluded (*n* = 27/200).

7. We also replaced some emotions included in the Pilot (i.e., Gratitude, Pride, Guilt) with the four frequently reported emotions among participants (i.e., Anxiety, Shock, Irritation, and Surprise), but there was no added value to including them in the present analyses.

8. In addition to measuring perceptions of people in their direct environment, we also measured these items with reference to the Dutch population in general (including the Polarization Index; see Supplementary Materials p. 9), but these are not relevant for the comparison of regional contexts and thus we report them elsewhere.

9. See Supplementary Materials (p. 11) for the statistical analysis plan and formal tests of assumptions.

10. We also tested models with perceived dyadic harm and immorality as mediators, which suggested full mediation for both variables. Importantly, however, these findings should be interpreted with caution, as we found medium-to-large-sized bivariate correlations among moral emotion, dyadic harm, and immorality (all *r* > .62, all *p* < .001; Cohen, 1992). Taken together, this suggests that although Hypothesis 2 is generally supported, alternative hypotheses regarding the mediating roles of perceived dyadic harm as well as perceived immorality cannot be rejected.

11. As in Experiment 1, we also tested for mediation models with perceived dyadic harm and immorality (all *r* > .66, all *p* < .001), which yielded similar conclusions.

12. Participants who failed the attention check (*n=* 55/550; 10%) were excluded.

13. For attenuated (cf. ordinal) interaction effects, the achieved power will be only 50% of the default cross-over interaction effects (Lakens & Caldwell, 2019). Therefore, we divided a medium effect size (*f* = 0.250) by two to arrive at a sensible required sample size.

14. In each of the three experiments, and as outlined in the Supplementary Materials on p. 12, we found an effect of the (strong) dyadic harm manipulation on punishment and also on social distance in Experiments 2 and 3. This is in line with the idea that moralization can be conceptualized as part of a broader value protection response.

15. Note that support for this interaction effect was also absent in Experiments 1 and 2 (see Supplementary Materials pp. 13–14), but these findings should be interpreted with caution because these studies were not designed to have sufficient statistical power to detect this effect.
