# Supplementary material for: Attitude Moralization Within Polarized Contexts: An Emotional Value-Protective Response to Dyadic Harm Cues
Source: Pers Soc Psychol Bull. 2021 Oct 5;48(11):1566–79. doi: 10.1177/01461672211047375 (PMC9548660; doi:10.1177/01461672211047375)
Supplement: sj-docx-1-psp-10.1177_01461672211047375 – Supplemental material for Attitude Moralization Within Polarized Contexts: An Emotional Value-Protective Response to Dyadic Harm Cues [file sj-docx-1-psp-10.1177_01461672211047375.docx]

Supplementary Materials

D’Amore, C., Van Zomeren, M., & Koudenburg, N. (2021). Attitude moralization within polarized contexts: An emotional value-protective response to dyadic harm cues. *Personality and Social Psychology Bulletin.*

**Table of Contents**

Demographic Information of the Sample for each Experiment (Incl. Table S1)…………….. 3

Pilot Experiment……………………………………………………………………………… 4

Table S2: Means (SD) by condition, and correlation matrix for all variables relevant to the manipulation of dyadic harm across experiments*………………*………………………..…….. 6

Additional Experimental Manipulation Checks Across Experiments ……………….………. 7

Additional Measures………………………………………………………………………….. 9

Statistical Analysis Plan……………………………………………………………………... 11

Converging Evidence: Social Distance and Need to Punish………………………………… 12

Additional Exploration of a Potential Amplification Effect of Perceived Polarization……... 13

Table S3: Means (*SD*) for individual perceptions of polarization and moralization *in the Netherlands* across experiments…………………………………………………………….. 14

Experimental Manipulation: Materials……………………………………………………… 15

References..…………………………………………………………………………………. 17

**Demographic Information of the Sample for Each Experiment**

| **Table S1.**  *Means (SD) for individual difference variables across experiments* | | | | |
| --- | --- | --- | --- | --- |
| Experiment | Attitude: Conservative | Political  Orientation | Moral conviction (pre-measure) | Moral conviction (post-measure) |
| Pilot (*N* = 66) | 2.68 (1.36) | 4.62 (1.91) | - | 2.45 (1.04) |
| 1 (*N* = 173) | 4.70 (0.46) | 5.24 (2.07) | 3.16 (1.09) | 3.12 (1.17) |
| 2 (*N* = 155) | 4.57 (0.50) | 5.83 (1.96) | 3.21 (1.02) | 3.24 (1.07) |
| 3 (*N* = 495) | 6.19 (0.86) | 5.93 (1.82) | 2.85 (1.23) | 2.92 (1.02) |
| Attitude (5-point scale in Pilot and Experiments 1-2; 7-point scale in Experiment 3; from 1 = very strongly agree to 5 (7) = very strongly disagree), political orientation (10-point scale from 1 = extremely left-winged to 10 = extremely right-winged), moral conviction (5-point scale, from 1 = not at all to 5 = completely). | | | | |

**Experiment 3: Geographic sample information**

The sample was representative for the twelve Dutch provinces: Groningen (*n* = 20, 4.0%), Friesland (*n* = 8, 1.6%), Drenthe (*n* = 14, 2.8%), Noord-Holland (*n* = 67, 13.5%), Overijssel (*n* = 29, 5.9%), Flevoland (*n* = 17, 3.4%), Gelderland (*n* = 59, 11.9%), Zuid-Holland (*n* = 122, 24.6%), Utrecht (*n* = 33, 6.7%), Noord-Brabant (*n* = 82, 16.6%), Zeeland (*n* = 8, 1.6%), Limburg (*n* = 36, 7.3%). The sample is also approximately representative regarding the degree of urbanization, which distinguishes rural areas (*n=* 121, 24.4%), small cities (*n=* 188, 38.0%) and large cities (*n=* 186, 37.6%).

**Pilot Experiment**

This pilot experiment had two specific aims. First, as converging evidence, we tested to what extent our developed manipulation of a conflict-prone (vs. harmonious) action approach successfully increased perceptions of dyadic harm and closely related perceptions of immorality. Additionally, we tested whether perceptions of dyadic harm and immorality are indeed strongly correlated (Schein & Gray, 2018). Second, we tested whether participants reported decreased support for the conflict-prone (vs. harmonious) outgroup approach, but not their aim (i.e., to change *Zwarte Piet*), which was held constant between conditions.

**Participants**

Participants were 66 Dutch first-year psychology students (74% female; *M*_age_= 20.35, *SD* = 3.35, range = 18–39), recruited through the psychology student panel of the University of Groningen.

**Measurements and Procedure**

The measures used for the main variables as well as the study procedure were similar to those reported in Experiments 1-3.

**Results and Discussion Pilot**

Table S2 provides a full overview of the results and variable statistics. As intended, the manipulation of the conflict-prone (vs harmonious) group action induced significantly increased perceptions of dyadic harm (*t*(64)= -4.53, *p* < .001, *d* = 1.11) and immorality (*t*(64) = -5.40, *p* < .001, *d* = 1.33), which are also strongly correlated (*r* = .76, *p* < .001), supporting their presumed interconnectedness. Moreover, as intended, the manipulation of the conflict-prone (versus harmonious) action elicited significantly decreased support for the outgroup’s action *approach* (*t*(64) = 10.80, *p* < .001, *d* = 2.66), and we found no significant differences regarding participants’ support for the group’s *aim* (*t*(64) = -1.43, *p* = .157, *d* = 0.35). Thus, the manipulation successfully manipulated the intended perceptions of dyadic harm, immorality, and reduced support for the approach, while no differences between support for the aim of the action group was found.^^[[1]](#footnote-1)^^

Although the manipulation had a large-sized effect on perceived dyadic harm (Cohen, 1992), the average score in the conflict-prone condition was still below the mid-point of the 5-point scale (*M* = 2.68). To increase this average score, we added a sentence in the manipulation text about their intention to hinder the national festivities (taking place throughout November 2019) specifically until no black face helpers would be present, which we assumed would elicit stronger perceptions of dyadic harm because of the increased anticipation of heated conflict in the presence of children at this event.

Additionally, the manipulation led to significantly increased experiences of negative moral emotion (*t*(64) = -2.53, *p* = .014, *d* = 0.62), as expected. However, this medium-sized effect was lower than expected, which may in part be due to its abstract phrasing (i.e., “*To what extent did you feel [emotion] when reading the news message*?”).^^[[2]](#footnote-2)^^ Therefore, we slightly adapted this measure by means of specifying the manipulated outgroup action as the target of experienced emotions (“*To what extent did you feel [emotion] towards this specific group and their statements*?”).

**Table S2**. Means (SD) by condition, and correlation matrix for all variables relevant to the manipulation of dyadic harm across experiments

|  | **Means (SD): *Conflict-prone*** | **Means (SD): *Harmonious*** | **2.** | **3.** | **4.** | **5.** | **6.** |
| --- | --- | --- | --- | --- | --- | --- | --- |
| **1. Dyadic Harm**  *Pilot*  *Experiment 1*  *Experiment 2*  *Experiment 3* | 2.86^b^ (0.73)  4.12^b^ (0.82)  3.97^b^ (0.97)  3.94^b^ (0.94) | 1.94^a^ (0.93)  3.13^a^ (1.24)  2.84^a^ (1.15)  2.93^a^ (1.14) | .78*  .71*  .82*  .73* | -.59*  -.26*  -.30*  -.62* | -.48*  .09  .09  -.50* | --  --  --  .59* | --  --  --  .63* |
| **2. Immorality**  *Pilot*  *Experiment 1*  *Experiment 2*  *Experiment 3* | 2.99^b^ (1.05)  4.31^b^ (0.79)  4.27^b^ (0.84)  4.26^b^ (0.85) | 1.65^a^ (0.98)  3.36^a^ (1.26)  2.94^a^ (1.21)  3.22^a^ (1.27) | --  --  --  -- | -.70*  -.36*  -.46*  -.70* | -.54*  .09  -.07  -.54* | --  --  --  .70* | --  --  --  .62* |
| **3. Support Approach**  *Pilot*  *Experiment 1*  *Experiment 2*  *Experiment 3* | 1.24^b^ (0.55) 1.48^b^ (1.19) 1.59^b^ (1.19) *6.59^b^ (0.87)* | 3.62^a^ (1.16)  2.33^a^ (1.30)  2.85^a^ (1.27)  *5.04^a^ (1.64)* |  | --  --  --  -- | .34*  .44*  .54*  .63* | --  --  --  -.78* | --  --  --  -.52* |
| **4. Support Aim**  *Pilot*  *Experiment 1*  *Experiment 2*  *Experiment 3* | 2.24^a^ (1.23) 1.49^a^ (1.16)  1.66^a^ (1.23)  *6.31^b^ (1.05)* | 2.69^a^ (1.33) 1.45^a^ (1.04) 1.82^a^ (1.24)  *5.77^a^ (1.33)* |  |  | --  --  --  -- | --  --  --  -.48* | --  --  --  -.58* |
| **5. Extremity Approach**  *Pilot*  *Experiment 1*  *Experiment 2*  *Experiment 3* | --  --  --  4.74^b^ (0.52) | --  --  --  3.00^a^ (1.50) |  |  |  | --  --  --  -- | --  --  --  .68* |
| **6. Extremity Aim**  *Pilot*  *Experiment 1*  *Experiment 2*  *Experiment 3* | --  --  --  4.46^b^ (0.86) | --  --  --  3.62^a^ (1.36) |  |  |  |  | --  --  --  -- |
|  |  |  |  |  |  |  |  |

*Note.* Means (SD) in regular font were measured on a 5-point scale (1 = not at all, 5 = completely). Means (SD) in italics were measured on a 7-point scale (1 = very strongly agree, 7 = very strongly disagree) and reversed coded. Means with different subscripts differ significantly from each other at *p* < .05. Correlation coefficients: **p* < .001.

**Additional Experimental Manipulation Checks across Experiments**

As in the Pilot, participants indicated their support for the manipulated protest aim and approach (“*Do you support the aim [approach] of the group’s planned action?*”). As expected, participants in the conflict-prone (versus harmonious) condition reported significantly lower support for the manipulated action approach in Experiment 1(*M_difference_*= 0.85, 95% CI [0.48, 1.23], *t*(171) = 4.53, *p* < .001, *d* = 0.69) and Experiment 2 (*M_difference_*= 1.26, 95% CI [0.87, 1.65], *t*(153) = 6.42, *p* < .001, *d* = 1.03). Additionally, no significant between-condition differences arose for support for the manipulated action aim in Experiment 1 (*M_difference_*= 0.04, 95% CI [-0.37, 0.29], *t*(171) = 0.24, *p* = .811, *d* = 0.04) and Experiment 2 (*M_difference_*= 0.16, 95% CI [-0.23, 1.66], *t*(153) = 0.82, *p* = .412, *d* = 0.13).

**Experiment 3**

To further investigate the validity of the manipulation, additional manipulation checks were assessed in Experiment 3: Participants completed two single-item measures assessing perceived extremity of the action approach and aim (i.e., “*To what extent do you think that the group’s action approach [aim] is extreme*”).

Table S2 provides an overview of the bivariate correlations between the variables relevant to the internal validity of our manipulation. Two relevant points are discussed here. First, large-sized correlations are found between perceptions of dyadic harm and immorality (*t*(493) = 23.42, *r* = .73, *p* < .001), which supports their expected interconnectedness and resembles the effect sizes found in Experiments 1-2. Second, Experiment 3 also shows large-sized correlations between the most relevant newly added manipulation variable, extremity of approach, and both dyadic harm and immorality (both *r* > .62, both *p* < .001), which is in line with our expectations.

As in Experiments 1-2, participants in the conflict-prone (vs. harmonious) condition also reported increased opposition against the (conflict-prone/harmonious) group approach (*M_difference_*= 1.54, 95% CI [1.31, 1.77], *t*(493) = 17.20, *p* < .001, *d* = 1.55). Additionally, participants in the conflict-prone (vs. harmonious) condition also perceived the group approach as more extreme (*M_difference_*= 1.74, 95% CI [1.54, 1.94], *t*(493) = 13.07, *p* < .001, *d* = 1.17). This suggests variables that should be closely related to perceived dyadic harm were indeed affected by the manipulation in the expected direction, which offers converging evidence for its validity.

Somewhat unexpectedly, in Experiment 3 participants in the conflict-prone (vs. harmonious) condition also reported significantly stronger opposition against aim (*M_difference_* = 0.54, 95% CI [0.33, 0.75], *t*(493) = 5.03, *p* < .001, *d* = 0.45) as well as extremity of aim (*M_difference_* = 0.84, 95% CI [0.64, 1.04], *t*(493) = 8.18, *p* < .001, *d* = 0.74). To investigate the alternative possibility that these effects may underlie the effect of the manipulation on perceptions of dyadic harm, multiple regression analyses were conducted. Specifically, both opposition against aim and extremity of aim were entered simultaneously in an ANCOVA with the conflict-prone (vs. harmonious) condition and perceived dyadic harm as outcome variable. In both cases, however, the effect of the conflict-prone (vs. harmonious) condition remained positive and significant, which suggests that this effect is not likely to be confounded by participants’ support for or perceived extremity of the group’s aim.

**Additional Measures**

**Variables related to Polarization**

**Polarization Index for opinion polarization.** As another indicator of polarization perceptions, we used the Polarization Index for opinion polarization (Koudenburg et al., 2021; also see Kusumi et al., 2017). Participants *distributed* the opinions of the Dutch public [their social environment] about the issue of *Zwarte Piet* in the following way: “*Imagine that we would ask 10 randomly selected Dutch people about their opinion towards the same statement. They can indicate their agreement with the same statement by selecting one of the options of the scale below. We would like you to estimate the opinions of these 10 Dutch people. For the statement ‘I oppose change in the traditional Zwarte Piet’, how many of these 10 people would … 1) strongly disagree, 2) disagree, 3) neither agree nor disagree, 4) agree, 5) strongly agree?*”. The perceived polarization index was calculated by using a weighted average of the level of disagreement between each pair of respondents from the distribution.^^[[3]](#footnote-3)^^

**Perceived Consensus** A single item was used to measure perceived consensus in the Netherlands and locally (“*In the Netherlands [my direct social environment], most people think the same about this issue*”). In Experiment 3, we added another item (“*Despite small differences, most people in the Netherlands [my direct social environment] have similar opinions about this issue*”) to make a 2-item scale (*a_(NL)_*= .82, *a_(local)_*= .82).

**Perceived Societal Moralization**. In a similar fashion as the measure for perceived societal polarization, participants distributed 10 randomly selected Dutch people, now regarding the extent to which their stance on the *Zwarte Piet* issue is a moral conviction. Perceived societal moralization was operationalized as the average score of the 10 Dutch people (i.e., the mean).

**Variables related to Value-Protective Responses**

**Need to Punish.** Participants indicated their need to punish the outgroup by indicating agreement with a self-developed set of three statements (i.e., “*The group’s planned action should be stopped* [*prevented*]”; “*The group should be punished for executing the planned action”*; *a_(Experiment 1)_* = .90, *a_(Experiment 2)_* = .92; *a*_(_*_Experiment 3_*_)_ = .91).

**Social distance.** Participants reported on the extent of desired social distance with attitudinal opponents concerning their stance on the issue of *Zwarte Piet*, by indicating their agreement (5-point scale, 1 = not at all, 5 = very much; *a_(Experiment 1)_* = .93, *a_(Experiment 2)_* = .88; *a*_(_*_Experiment 3_*_)_ = .89), with two statements (“*I would find it difficult to be friends with someone who has a dissimilar stance as me on this issue*”; “*I would feel a certain distance if I would find out that the person I interact with thinks differently about the issue*”; Koudenburg & Kashima, 2021)^[[4]](#footnote-4)^.

**Ruining the Celebration.** In Experiment 3, we added a measure at the end of the survey that assesses the extent to which participants believed the children’s celebration would be ruined by the group and their planned actions. Participants reported on the degree to which they believed that the celebration would be ruined by the group and their planned actions (3-item measure; “*The group and their planned actions are ruining the children’s celebration.*”; “*The children will become the victims of the group and their planned actions.*”; *The activists of the group want to ruin the children’s celebration with their planned actions.*”; *a* = .90).

**Statistical Analysis Plan**

We were mainly interested in testing within-subject changes in moral conviction scores (i.e., *moralization*) as a function of the conflict-prone (vs. harmonious) group condition. Although it is possible to test our Hypothesis 1-2 by conducting an ANOVA with moral conviction difference scores (MC_post_ - MC_pre_) as dependent variable, increased statistical power is achieved when conducting an ANCOVA with post-scores as the dependent variable and pre-scores as a covariate (e.g., Johnson. 2016; Tabacknick & Fidell, 2012). Importantly, this ANCOVA approach is valid when the slopes of the regression lines for the two experimental groups are equal. In all three experiments, ANCOVAs suggested no evidence for a violation of the assumption of equal regression slopes. Specifically, there was no significant interaction between MC_pre_ scores and the experimental condition in Experiment 1 (*F*(1,169) = 1.516, *p* = .220), Experiment 2 (*F*(1,151) = 0.126, *p* = .724), and Experiment 3 (*F*(1,491) = 0.102, *p* = .750). We thus conducted ANCOVAs with MC_post_ as the outcome and MC_pre_ as the covariate to test Hypotheses 1-2, and we added MC_pre_ as a covariate in each of the models tested under the explorative analyses. This allowed us to further explore the effects of the conflict-prone (versus harmonious) group manipulation (i.e., on additional outcomes), independent of pre-manipulation between-condition differences in moral conviction scores.

**Converging Evidence of Value-protective Responses: Social Distance and Punishment**

**Need to Punish.** Across all three experiments, ANCOVAs indicated a significant between-condition difference on need to punish (*Exp 1*: *F*(1,170) = 35.974, p < .001; *Exp 2*: *F*(1,152) = 65.64, *p* < .001; *Exp 3*: *F*(1,492) = 165.14, *p* < .001): as expected, participants in the conflict-prone (versus harmonious) condition indicated a stronger need to punish the outgroup (*Exp 1*: *b* = 1.00, 95% CI[0.67, 1.32], large-sized effect, ${}_{p}^{2}$ = .175; *Exp 2*: *b* = 1.45, 95% CI[1.10, 1.80], large-sized effect, ${}_{p}^{2}$= .302; *Exp 3*: (*b* = 1.25, 95% CI[1.06, 1.44], large-sized effect,${}_{p}^{2}$ = .251).

**Social Distance.** In Experiment 1, we found no significant between-condition differences on social distance (*F*(1,170) = 0.079, *p* = .779). However, in Experiment 2 and 3, an ANCOVA indicated a significant between-condition difference on social distance (*Exp 2*: *F*(1,152) = 4.350, *p* = .039; *R^2^_adjusted_* = .20; *Exp 3*: *F*(1,492) = 11.20, *p* < .001; *R^2^_adjusted_* = .15). In line with our expectations, regression analyses revealed that participants in the conflict-prone (versus harmonious) condition indicated an increased desire for social distance towards attitudinally different others (*Exp 2*: *b* = 0.41, 95% CI [0.02; 0.80], *t*(152) = 2.086, ${}_{p}^{2}$ = .03; *Exp 3*: *b* = 0.36, 95% CI [0.15; 0.57], *t*(492) = 3.347, ${}_{p}^{2}$ = .02). Thus, in line with the effect found for moralization, this suggests that the manipulation of the conflict-prone (versus harmonious) group lead participants to increase their desire for social distance towards attitudinal opponents in general.^^[[5]](#footnote-5)^^

**Additional Exploration of a Potential Amplification Effect of Perceived Polarization**

Because there was no meaningful difference between Experiments 1 and 2 in terms of perceived polarization^[[6]](#footnote-6)^, but there is substantial variation at the individual level in terms of perceived polarization within each experiment, we conducted the same interaction analyses with individual perceptions as in Experiment 3 to provide a complete overview of the potential interaction effects across all studies (see Table S3 for Means (SD) across experiments). It is important to note, however, that experiments 1 and 2 were not designed to have enough statistical power to properly perform these interaction analyses. Hence, the findings reported below for experiment 1 and 2 should be interpreted with caution.

**Experiment 1.** A mixed-effects model with subjects at Level 2 and time (pre-measure and post-measure moral conviction) at Level 1 were conducted to test for potential interaction between perceived polarization in society and the *conflict-prone* (vs. *harmonious*) group condition on within-subject moralization, but no support was found for this interaction (*F*(1,169) = 0.013, *p* = .910). Also no effects were found for perceived polarization locally (*F*(1,169) = 2.236, *p* = .137). Additionally, no interaction effects were found for the related perceptions of *consensus* in society (*F*(1,169) = 0.260, *p* = .611) or locally (*F*(1,169) = 0.462, *p* = .500), and the opinion Polarization Index in society (*F*(1,169) = 0.013, *p* = .910).

**Experiment 2.** Similar to Experiment 1, a mixed-effects model showed no support for an interaction between perceived polarization and the *conflict-prone* (vs. *harmonious*) group condition on within-subject moralization (*F*(1,151) = 0.042, *p* = .838). Also no effects were found for perceived polarization locally (*F*(1,151) = 0.834, *p* = .363). Additionally, again, no interaction effects were found for the related perceptions of *consensus* in society (*F*(1,151) = 0.644, *p* = .423) or locally (*F*(1,151) = 2.751, *p* = .100), and the opinion Polarization Index in society (*F*(1,151) = 0.130, *p* = .719).

**Experiment 3.** As in Experiments 1-2, mixed-effect models did not indicate significant interaction effects with the *conflict-prone* (vs. *harmonious*) group condition on within-subject moralization for between perceived polarization in society (*F*(1,491) = 0.512, *p* = .475) or local (*F*(1,491) = 0.648, *p* = .421), perceived Polarization Index in society (*F*(1,491) = 0.114, *p* = .736); perceived consensus in society (*F*(1,491) = 1.246, *p* = .265) and local (*F*(1,491) = 0.568, *p* = .444).

**Amplification effect of perceived societal moralization (Experiment 3)**

For participants’ perceptions of *societal moralization* in the Netherlands in general, we found a significant interaction effect for with the conflict-prone (vs. harmonious) group condition on moralization (*F*(1,490) = 3.952, *p* = .047). Simple slope analysis revealed that for participants who perceived *high* societal moralization (+1*SD*), the conflict-prone action lead to significantly stronger moralization as compared to the harmonious action (*B* = 0.32, 95% CI [0.11, 0.52], *t*(490) = 3.057, *p* < .005), whereas for participants who perceived *low* societal moralization (-1*SD*) no significant difference in moralization was found between the two experimental conditions (*B* = 0.03, 95% CI [-0.18, 0.23], *t*(490) = 0.244, *p* = .807).

**Table S3.** Means (*SD*) for individual perceptions of polarization and moralization *in the Netherlands* across experiments

| Experiment | Perceived Polarization | Perceived (opinion) Polarization Index | Perceived Consensus | Perceived Moralization |  |
| --- | --- | --- | --- | --- | --- |
| 1 | 3.58 (0.96) | 0.31 (0.22) | 3.53 (1.16) | 3.50 (0.93) |  |
| 2 | 3.66 (0.85) | 0.34 (0.23) | 3.30 (1.22) | 3.35 (0.94) |  |
| 3 | 3.72 (0.73) | 0.36 (0.22) | 3.08 (1.29) | 3.40 (1.02) |  |
| *Note.* All variables were measured on a 5-point scale, except for Perceived Polarization Index (see “Additional Measures”, p.9). | | | | |  |

**Experimental Manipulation: Materials**

**Conflict-prone condition**


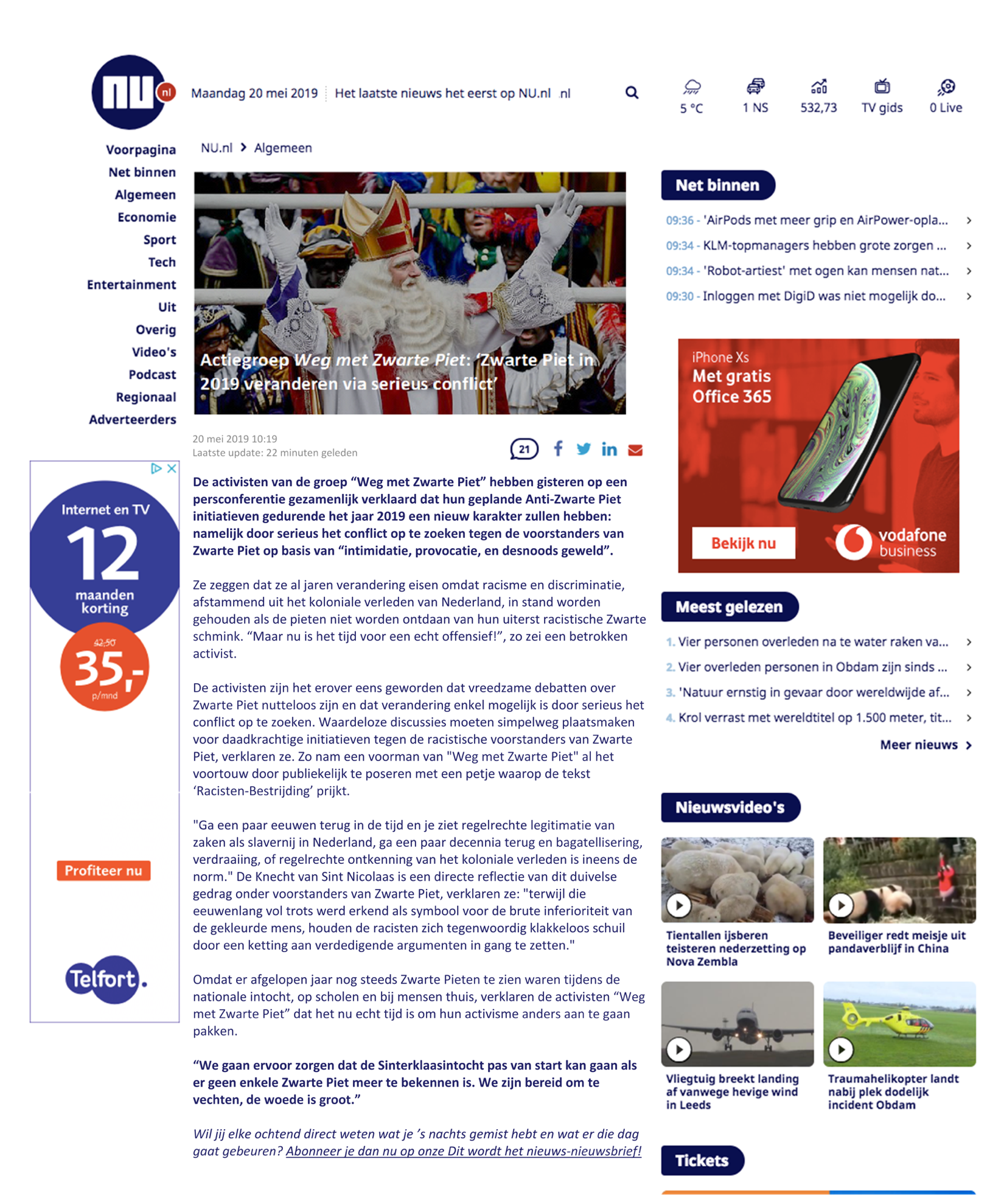


**Harmonious condition**


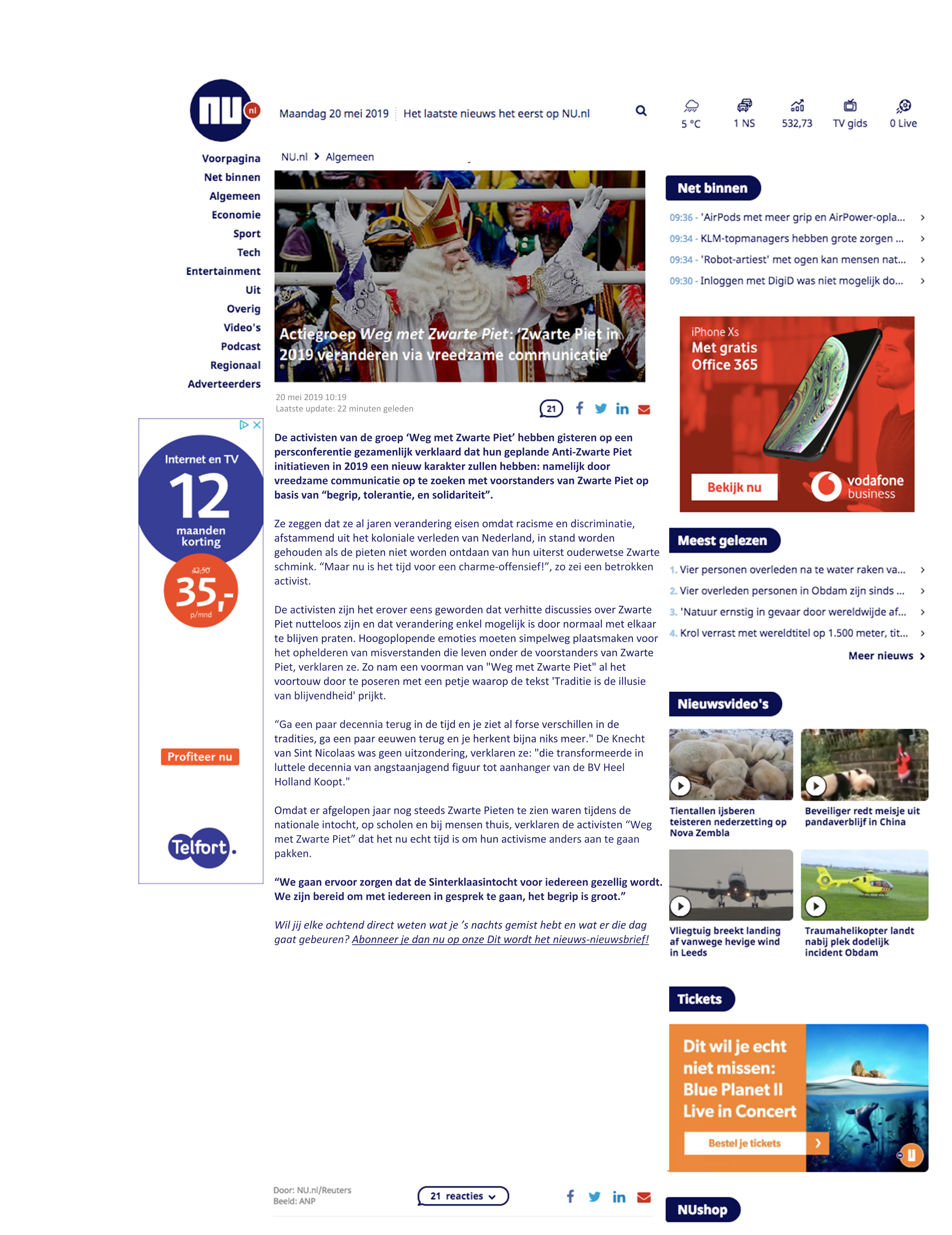


References

Johnson, T. R. (2016). Violation of the homogeneity of regression slopes assumption in ANCOVA for two-group pre-post designs: Tutorial on a modified Johnson-Neyman procedure. The Quantitative Methods for Psychology, 12(3), 253– 263. doi:10.20982/tqmp.12.3.p253

Henrich, J., Heine, S. J., & Norenzayan, A. (2010). The weirdest people in the world?. *Behavioral and brain sciences*, *33*(2-3), 61-83.

Tabachnick, B. G. & Fidell, L. S. (2012). Using multivariate statistics (6th ed.) Boston, MA: Pearson/Allyn & Bacon.

Koudenburg, N., & Kashima, Y. (2021). A Polarized Discourse: Effects of Opinion Differentiation and Structural Differentiation on Communication. *Personality and Social Psychology Bulletin*. <https://doi.org/10.1177/01461672211030816>

Koudenburg, N., Kiers, H. A. L., & Kashima, Y. (2021). A new Opinion Polarization Index developed by integrating expert judgments*. Frontiers in Psychology*. doi: 10.3389/fpsyg.2021.738258.

Kusumi, T., Hirayama, R., & Kashima, Y. (2017). Risk perception and risk talk: The case of the Fukushima Daiichi nuclear radiation risk. *Risk Analysis*, *37*(12), 2305-2320. doi:10.1111/risa.12784

Wisneski, D. C., & Skitka, L. J. (2017). Moralization through moral shock: Exploring emotional antecedents to moral conviction. *Personality and Social Psychology Bulletin, 43*(2), 139-150. doi:10.1177/0146167216676479

1. The effect sizes of this pilot study might be biased, as they are based on a sample of psychology students who tend to endorse relatively liberal rather than conservative stances on political issues (Henrich, Heine, & Norenzayan, 2010). Even so, this would imply that the effect sizes will likely be stronger (rather than weaker) in the following studies that pre-selected participants with a conservative stance on this specific issue. [↑](#footnote-ref-1)
2. See Wisneski & Skitka, 2017, for a discussion on the importance of measuring *integral-* as opposed to *incidental affect*. [↑](#footnote-ref-2)
3. Difference pairs [1-2;2-3;3-4;4-5;1-3;3-5] receive no weight, pair [2-4] receives 1.07 weight, pairs [1-4;2-5] receive 1.35 weight, pair [1-5] receives 1.98 weight. These weights produce a Polarization Index that is related to the standard deviation, but more sensitive to the bimodality of the distribution (Koudenburg et al., 2021). [↑](#footnote-ref-3)
4. For Experiment 3, we added a third item to this previously two-item measure (i.e., “*I would feel comfortable with having close friends who have an opposite opinion on the issue*”). However, this item decreased the alpha coefficient of this scale from .88 to .70. We therefore removed this item from this scale again. [↑](#footnote-ref-4)
5. Again, bootstrap mediation tests suggested that the mediating role of 1) negative moral emotion, 2) perceived dyadic harm, and 3) perceived immorality on need for sanctioning and social distance are certainly not rejected in Experiments 1-3 (although we did not find support for a direct effect on social distance in Experiment 1). [↑](#footnote-ref-5)
6. Note that on a different measure, namely *perceived consensus*, independent sample t-tests showed a significant difference: participants in Experiment 1 perceived significantly more consensus on the *Zwarte Piet* issue among people in their direct social environment (*M* = 4.24, *SD* = 0.94) compared to participants in Experiment 2 (*M* = 4.02, *SD* = 1.01; *t*(326) = 2.02, *p* = .044, *d* = 0.22). [↑](#footnote-ref-6)
