# Supplementary material for: Attitude Moralization Within Polarized Contexts: An Emotional Value-Protective Response to Dyadic Harm Cues
Source: Pers Soc Psychol Bull. 2021 Oct 5;48(11):1566–79. doi: 10.1177/01461672211047375 (PMC9548660; doi:10.1177/01461672211047375)
Supplement: sj-docx-2-psp-10.1177_01461672211047375 – Supplemental material for Attitude Moralization Within Polarized Contexts: An Emotional Value-Protective Response to Dyadic Harm Cues [file sj-docx-2-psp-10.1177_01461672211047375.docx]

| Variable name (R-script) | Variable name (manuscript) | # Items | Code |
| --- | --- | --- | --- |
| Cond | Experimental condition | NA | 0 = Harmonious (weak dyadic harm)  1 = Conflict-prone (strong dyadic harm) |
| MC1 | Moral conviction (pre) | 4 | Ranges from 1 to 5; higher scores indicate stronger moral conviction |
| MC2 | Moral conviction (post) | 4 | Ranges from 1 to 5; higher scores indicate stronger moral conviction |
| DH | Perceived dyadic harm | 3 | Ranges from 1 to 5; higher scores indicate stronger perceptions |
| NegMo | Negative moral emotion | 3 | Ranges from 1 to 5; higher scores indicate stronger emotions |
| Im | Perceived Immorality | 3 | Ranges from 1 to 5; higher scores indicate stronger perceptions |
| EntrNL  (Exp 3) | Perceived structural polarization | 5 | Ranges from 1 to 5; higher scores indicate stronger perceptions |

Main variables

Additional variables

| Variable name (R-script) | Variable name | # Items | Code |
| --- | --- | --- | --- |
| MCdiff | Moral conviction difference (post-pre) | 8 | Ranges from -5 to 5; higher scores indicate stronger increase in moral conviction post-manipulation |
| Att | Attitude | 1 | Ranges from 3-5; higher scores indicate stronger attitude opposing change in the traditional Zwarte Piet |
| UrRu | Urbanization of place of residence | 1 | 1 = Big city (> 100.000 residents)  2 = Small city (< 100.000 residents)  3 = Village or countryside |
| RelThr | Social distance | 2 | Ranges from 1 to 5; higher scores indicate stronger desire for social distance |
| Threat | Punishment | 3 | Ranges from 1 to 5; higher scores indicate stronger desire for punishment |
| PODG23  (Exp 1-2) | Perceived structural polarization | 2 | Ranges from 1 to 5; higher scores indicate stronger perceptions |
| PODL23  (Exp 1-2) | Perceived structural polarization local | 2 | Ranges from 1 to 5; higher scores indicate stronger perceptions |
| EntrL  (Exp 3) | Perceived structural polarization local | 5 | Ranges from 1 to 5; higher scores indicate stronger perceptions |
| PODG1  (Exp 1-2) | Perceived consensus NL | 1 | Ranges from 1 to 5; higher scores indicate stronger perceptions |
| ConsNL  (Exp 3) | Perceived consensus NL | 2 | Ranges from 1 to 5; higher scores indicate stronger perceptions |
| PODL1  (Exp 1-2) | Perceived consensus local | 1 | Ranges from 1 to 5; higher scores indicate stronger perceptions |
| ConsL  (Exp 3) | Perceived consensus local | 2 | Ranges from 1 to 5; higher scores indicate stronger perceptions |
| PpInd  (Exp 1-2) | Polarization (opinion) index | 5 | Difference pairs [1-2;2-3;3-4;4-5;1-3;3-5] receive no weight, pair [2-4] receives 1.07 weight, pairs [1-4;2-5] receive 1.35 weight, pair [1-5] receives 1.98 weight. These weights produce a Polarization Index that is related to the standard deviation, but more sensitive to the bimodality of the distribution. |
| PpIndNL  (Exp 3) | Polarization (opinion) index | 5 | Difference pairs [1-2;2-3;3-4;4-5;1-3;3-5] receive no weight, pair [2-4] receives 1.07 weight, pairs [1-4;2-5] receive 1.35 weight, pair [1-5] receives 1.98 weight. These weights produce a Polarization Index that is related to the standard deviation, but more sensitive to the bimodality of the distribution. |
| MCPODmean  (Exp 1-2) | Perceived moralization | 5 | Ranges from 1 to 5; higher scores indicate stronger perceptions |
| MCPODmeanNL  (Exp 3) | Perceived moralization | 5 | Ranges from 1 to 5; higher scores indicate stronger perceptions |
| Aim  (Exp 1-2) | Support for aim | 1 | Ranges from 1 to 5; higher scores indicate stronger support |
| App  (Exp 1-2) | Support for approach | 1 | Ranges from 1 to 5; higher scores indicate stronger support |
| Aim  (Exp 3) | Support for aim | 1 | Ranges from 1 to 7; higher scores indicate stronger support |
| App  (Exp 3) | Support for approach | 1 | Ranges from 1 to 7; higher scores indicate stronger support |
| AimEx  (Exp 3) | Extremity aim | 1 | Ranges from 1 to 5; higher scores indicate stronger extremity |
| AppEx  (Exp 3) | Extremity approach | 1 | Ranges from 1 to 5; higher scores indicate stronger extremity |
| PolOr | Political orientation | 1 | Ranges from 1 to 10; higher scores indicate stronger political conservatism |
| Childr  (Exp3) | Ruining the celebration | 3 | Ranges from 1 to 5; higher scores indicate stronger agreement |

Note: Some variables are included twice, once in its original form (see table above) and once in its standardized form. Variables ending with “z” are standardized around the mean (e.g., “EntrNLz” = “EntrNL” standardized), and are used for interaction analyses.
